# Performance variability in perioperative sentinel events: report on a nationwide data set

**DOI:** 10.1093/bjs/znac067

**Published:** 2022-04-04

**Authors:** Iris M. Reijmerink, Kelly Bos, Ian P. Leistikow, Jop Groeneweg, Fokie Cnossen, Dave A. Dongelmans, Maarten J. van der Laan

**Affiliations:** Impulse Institute, Amsterdam, the Netherlands; Department of Surgery, University Medical Centre Groningen, Groningen, the Netherlands; Impulse Institute, Amsterdam, the Netherlands; Department of Surgery, Amsterdam University Medical Centres—location Academic Medical Centre, Amsterdam, the Netherlands; Erasmus School of Health Policy and Management, Erasmus University, Rotterdam, the Netherlands; Dutch Health and Youth Care Inspectorate, Utrecht, the Netherlands; Impulse Institute, Amsterdam, the Netherlands; Centre for Safety in Healthcare, Delft University of Technology, Delft, the Netherlands; Unit of Cognitive Psychology, Leiden University, Leiden, the Netherlands; Impulse Institute, Amsterdam, the Netherlands; Department of Artificial Intelligence, Bernoulli Institute of Mathematics, Computer Science and Artificial Intelligence, University of Groningen, Groningen, the Netherlands; Impulse Institute, Amsterdam, the Netherlands; Department of Intensive Care Medicine, Amsterdam University Medical Centres—location Academic Medical Centre, Amsterdam, the Netherlands; Impulse Institute, Amsterdam, the Netherlands; Department of Surgery, University Medical Centre Groningen, Groningen, the Netherlands

## Abstract

This study aimed to evaluate how performance variability contributes to perioperative sentinel events occurring within the Dutch healthcare system. Although performance variability was identified as a contributing factor in almost all analysis reports, it remains unrecognized by the sentinel event analysis team. Strengthening the integration of performance variability in sentinel event analyses could improve the analyses performed, and lead to more effective improvement measures specifically aimed at optimizing human performance within the healthcare system.

## Introduction

Sentinel events are unintended events causing death or serious harm to patients, and these remain widespread in healthcare across the globe^1,2^. Healthcare organizations in many countries are mandated to report sentinel events to national reporting systems, analyse them to determine root causes, and develop improvement measures to prevent recurrence^3,4^. Although the quality of sentinel event analysis has increased, similar events keep recurring^4,5^. Research suggests this might be due to the quality of the improvement measures^4,6^.

The capacity to develop effective improvement measures is impaired when sentinel event analyses overlook relevant contributing factors^7^. One such factor is performance variability^4^. Performance variability is the positive or negative variation in behaviour and performance caused by environmental, organizational, and work-related factors, as well as human and individual characteristics^8,9^. Reduction in performance variability is an important goal of quality management in industrial production and laboratory measurement^10,11^. Performance variability also has a profound impact on quality of care, but the integration of this as a factor in healthcare quality management remains scant^12,13^. Previously, Dutch hospitals have indicated that the teams performing sentinel event analyses do not usually have specific training or knowledge on performance variability, although the literature shows that this could contribute to addressing such variability adequately within a sentinel event analysis^4^.

The aim of this study was to establish how performance variability contributes to perioperative sentinel events, by examining how often performance variability is included in sentinel event analysis reports and forms the basis for suggested improvement measures.

## Methods

Ethical approval was not required for this study. All Dutch perioperative sentinel event analysis reports from July 2017 to July 2018 in the national database of the Dutch Health and Youth Care Inspectorate were analysed.

Whether performance variability was identified as a contributing factor to the sentinel event by the analysis team was first established, by examining whether the report explicitly mentioned performance variability or its synonyms (human factor(s), human error(s)). If not mentioned explicitly, performance variability as a factor contributing to the event was examined using the Human Factors Investigation Tool^14^. This tool identifies three levels in incidents caused by human factors: action errors occurring immediately before the incident (level 3), the thought processes leading to the action error (level 2), and the underlying causes (level 1). Performance variability was identified as a contributing factor if all three levels were found in the analysis report. If all three levels were not found, it was then established whether solely technical errors were stated as the cause of the sentinel event. Whether performance variability tools or literature were used in the analysis of the sentinel event was also analysed.

Finally, the suggested improvement measures were analysed to establish whether these addressed any performance shaping factors (PSFs) that may underlie performance variability. For this, the Standardized Plant Analysis Risk—Human Reliability Analysis method was used, which looks at eight PSFs^15^. Improvement measures were scored as good, adequate or insufficient, based on whether they addressed PSFs explicitly (good) or indirectly (adequate), or not at all (insufficient) (*Appendix S1*).

## Results

In total, 115 perioperative sentinel event analysis reports containing 442 improvement measures were evaluated. Characteristics of sentinel events are shown in *Table S1*. In two reports (1.7 per cent), the emergence of the sentinel event was considered being a result of technical errors only. None of the reports explicitly mentioned performance variability or its synonyms. In 113 reports (98.3 per cent), however, performance variability was identified as a contributing factor in the emergence of the sentinel event. Only one analysis report referred to performance variability literature, and none of the reports used performance variability tools in their analyses.

Of the 442 improvement measures, 12 (2.7 per cent) addressed one or more PSFs explicitly and were thus scored as good. Some 225 (50.9 per cent) addressed one or more PSFs indirectly and were therefore scored as adequate. Finally, 205 improvements (46.4 per cent) were scored as insufficient as they did not mention any PSFs (*Table 1*).

**Table 1:** Quality scores and examples of improvement measures from Dutch perioperative sentinel event analysis reports between July 2017 and July 2018

Improvement measure score	No. of measures (*n* = 442)	Example
**Good**	12 (2.7)	‘Introduction of a whiteboard in the operating room for writing down protheses sizes to prevent implanting the wrong prosthesis’ Scored as good because directly aimed at reducing the complexity of the task as the mental effort required to memorize the prothesis sizes is eliminated
**Adequate**	225 (50.9)	‘Changing the introduction program for new employees’ Although this measure was probably aimed at improving the experience and training of the operator(s) involved in the task, it did not mention what exactly needed to be changed nor the measured effect
**Insufficient**	205 (46.4)	‘to take appropriate action’

Values in parentheses are percentages.

Improvement measures addressing PSFs were mostly aimed at the formal procedures present (94 measures), ergonomics and human-machine interaction (62), and work processes (36) (*Table 2* and *Fig. S1*). For 20 improvement measures (4.5 per cent), it was unclear what PSF a measure was aimed to shape. As an example, ‘the integration of a short time-out moment when an operation takes longer than expected’, could be aimed at the work processes, the complexity of the task or enhancing communication within the operating team.

**Table 2:** Frequency and examples of performance shaping factors addressed in improvement measures from Dutch perioperative sentinel event analysis reports between July 2017 and July 2018

PSF	No. of measures covered by PSF (*n* = 442)	Example
**Available time to diagnose and act upon abnormal situation**	1 (0.2)	‘The on-call surgeon will preferably not be running the outpatient clinic simultaneously in order to provide sufficient supervision to the Emergency department’
**Stress and stressors**	3 (0.7)	‘Minimize phone use, and thus possible disturbance, during surgery’
**Complexity of task at hand**	2 (0.5)	‘From now on, patients with an anatomical variation will be operated by two surgeons instead of one’
**Experience and training of operator(s) involved in task**	19 (4.3)	‘Provide additional training in the recognition of complications following interventional cardiology procedures’
**Formal procedures present for task**	94 (21.3)	‘Developing a checklist for pacemaker implementation’
**Ergonomics and human–machine interaction**	62 (14.0)	‘Make a clearer distinction between the oxygen and medical air flow meter, by using a different type of connection for the access points to prevent mismatch’
**Physical and mental fitness for duty**	0 (0)	–
**Work processes**	36 (8.1)	‘Incorporating an additional moment of verification during the time-out, to confirm that a particular procedure is known to all those present’
**Unclear**	20 (4.5)	–

Values in parentheses are percentages. PSF, performance shaping factor.

## Discussion

Although performance variability was identified as an important contributing factor in almost all sentinel events, none of the analysis reports explicitly mentioned performance variability nor a synonym, and performance variability was under-represented in the improvement measures. These findings are alarming, as reducing performance variability is an important goal of quality management, and omitting it from improvement measures can therefore lead to suboptimal patient safety^10,11^.

Most of the improvement measures that addressed performance variability were aimed at procedures and work processes rather than at individual PSFs, such as the experience and training or physical and mental fitness of the operators. This resonates with the literature on root cause analysis, a frequently used approach to determine the cause of a sentinel event^16^. The root cause analysis literature suggests that analyses should focus primarily on systems and processes and not on individual performance, as system-level improvements are more effective^6^. Sentinel event analysis teams might also feel uncomfortable addressing individual performance issues, as this can be interpreted as blaming and shaming. Previous research, however, showed that individual and organizational factors contribute equally to the development of sentinel events. Thus, both must be considered in order to truly understand the nature of sentinel events^13^. The Royal College of Surgeons of England mortality and morbidity meeting has already started to consider this, with questions being asked whether system, patient or staff factors contributed to an adverse event^17^. Recognition of staff-related PSFs, such as stress, does not equate to blaming individuals, but creates opportunities to address the underlying causes of such PSFs.

Although the present results have shown that performance variability plays an important role in nearly all perioperative sentinel events, knowledge of performance variability was underutilized in the reports. This confirms earlier studies in the healthcare sector reporting that specific knowledge or training in performance variability is scarce in sentinel event analysis teams and quality and safety departments of hospitals^4,18^. Knowledge of performance variability is of value in understanding why a sentinel event occurred, as well as in redesigning healthcare processes and systems to reduce the chances of recurrence. Understanding how the work environment influences human performance could also lead sentinel event analysis to address the causative factors, and may thus lead to more effective improvement measures.

The main goal of integrating performance variability knowledge into sentinel event analyses should, however, not solely be to identify causes of the event. It can also help understand things that go right: the safety II paradigm^19^. Integrating performance variability into sentinel event analyses will help to appreciate the conditions that strengthen the ability to function well despite difficult circumstances^20^. Explicitly addressing performance variability could improve sentinel event analyses, lead to more effective improvement measures that optimize human performance in the healthcare system, therefore having a larger potential to reduce preventable harm.

## Supplementary Material

znac067_Supplementary_Data
