# Supplementary material for: Performance variability in perioperative sentinel events: report on a nationwide data set
Source: Br J Surg. 2022 Apr 4;109(7):573–5. doi: 10.1093/bjs/znac067 (PMC10364676; doi:10.1093/bjs/znac067)
Supplement: znac067_Supplementary_Data [file znac067_supplementary_data.zip › Supplementary_Appendix_1.docx]

**Appendix S1: Supplementary methods**

Ethical approval was not required for this study.

**Data collection**

Two investigators (KB, AFT) jointly screened all sentinel events reported in the national database of the Dutch Health and Youth Care Inspectorate between July 2017 and July 2018, and selected all perioperative sentinel events. Any discrepancies between the investigators were solved through discussion.

A perioperative sentinel event was defined as an event occurring before, during or after an intervention performed in the operating theatre or interventional suite by a medical doctor and an anaesthesiologist. All reports included had to have been closed by the Inspectorate since active cases could not be analysed.

**Data analysis**

Two investigators (KB, AFT) separately collected the following data from the analysis reports: sex and age of the patient; outcome of the sentinel event (major injury or death); type of healthcare organisation; department and medical specialty involved; whether performance variability was a contributing factor in the emergence of the event; and the use of literature or tools from the performance variability field. Collected data were compared, and any discrepancies were resolved through discussion between the two investigators.

One investigator (IMR) furthermore analysed the suggested improvement measures for the presence of performance variability. A second investigator (KB) verified a random 20% sample. Results from both investigators were discussed. As no major errors or differences were recognized, no further verification was undertaken.

**Data tools**

Two tools were used to investigate the presence of performance variability in the analysis reports and the suggested improvement measures. The first tool used is the Human Factors Investigation Tool (HFIT), a tool initially developed to investigate the impact of performance variability in the emergence of incidents in the UK offshore oil and gas industry.^14^ According to the HFIT, incidents are seen as the product of several sequential human factors causes, organized into three different levels: action errors, the behaviours immediately prior to the incident (level 3); situation awareness, the thought processes leading to the action error (level 2); and threats, the underlying causes that can encourage the occurrence of errors (level 1). The second performance variability tool is the Standardized Plant Analysis Risk-Human Reliability Analysis (SPAR-H) method.^15^ The SPAR-H method aims to identify PV by addressing both the harmful and beneficial influences of so-called performance shaping factors (PSFs), factors that affect performance. The SPAR-H method identifies eight Performance Shaping Factors (PSFs): Available time to diagnose and act upon an abnormal situation; Stress and stressors, namely the level of undesirable conditions and circumstances, that impede the operator from easily completing the task; Complexity of the task at hand, namely how difficult the task is to perform in the given context; Experience and training of the operator(s) involved in the task; Formal procedures present for the task at hand; Ergonomics and human machine interaction; Physical and mental fitness for duty, not related to training, experience or stress; and, Work processes, referring to all aspects of doing work, such as work culture and communication between colleagues.

**Performance variability in the analysis reports**

First, whether performance variability was identified as a contributing factor to the sentinel event by the analysis teams was established, by examining whether the analysis reports explicitly mentioned performance variability or its synonyms (i.e., human factor(s), human error(s)).

If performance variability was not explicitly mentioned, next it was examined whether performance variability as a contributing factor to the sentinel event was recognized using the HFIT (yes/no). Performance variability as a contributing factor was established if all three levels of an incident according to the HFIT were present in the analysis report: action errors, situation awareness and threats.^14^

In the analysis reports that did not mention performance variability, it was then examined whether solely technical errors were stated as the cause of the event in the analysis reports.

Furthermore, whether literature or tools from the performance variability field was used by the sentinel event analysis team.

**Performance variability in the improvement measures**

Whether the suggested improvement measures addressed performance variability was established using the SPAR-H method, by examining whether the improvement measures addressed any PSFs that may underlie performance variability. Improvement measures were scored as “good” if one or more of the PSFs were explicitly addressed, as “adequate” if one or more of the PSFs were indirectly addressed, and as “insufficient” if no PSFs were addressed by the improvement measures.

**Statistical analysis**

Descriptive statistics were used to summarize the data. Descriptive data were reported as numbers (n) and percentages (%) using IBM SPSS Statistics for Windows (version 26.0, IBM, Armonk, New York, USA).
