# Supplementary material for: Performance variability in perioperative sentinel events: report on a nationwide data set
Source: Br J Surg. 2022 Apr 4;109(7):573–5. doi: 10.1093/bjs/znac067 (PMC10364676; doi:10.1093/bjs/znac067)
Supplement: znac067_Supplementary_Data [file znac067_supplementary_data.zip › Supplementary_Table_1.docx]

**Table S1: Sentinel event characteristics**

| **Variable** | **Percentage of sentinel events** |
| --- | --- |
| Department:   - Operating room - Clinic or outpatient clinic - Intensive care - Emergency department | - 74% (n = 85) - 16% (n = 19) - 7% (n = 8) - 3% (n = 3) |
| Medical specialty:   - General surgery - Cardiothoracic surgery - Urology - Orthopaedics - Neurosurgery - Gynaecology - Anaesthesiology - Other | - 43% (n = 50) - 11% (n = 13) - 8% (n = 10) - 8% (n = 9) - 7% (n = 8) - 6% (n = 7) - 5% (n = 6) - 10% (n = 12) |
