# Supplementary figures and images for: Performance variability in perioperative sentinel events: report on a nationwide data set
Source: Br J Surg. 2022 Apr 4;109(7):573–5. doi: 10.1093/bjs/znac067 (PMC10364676; doi:10.1093/bjs/znac067)

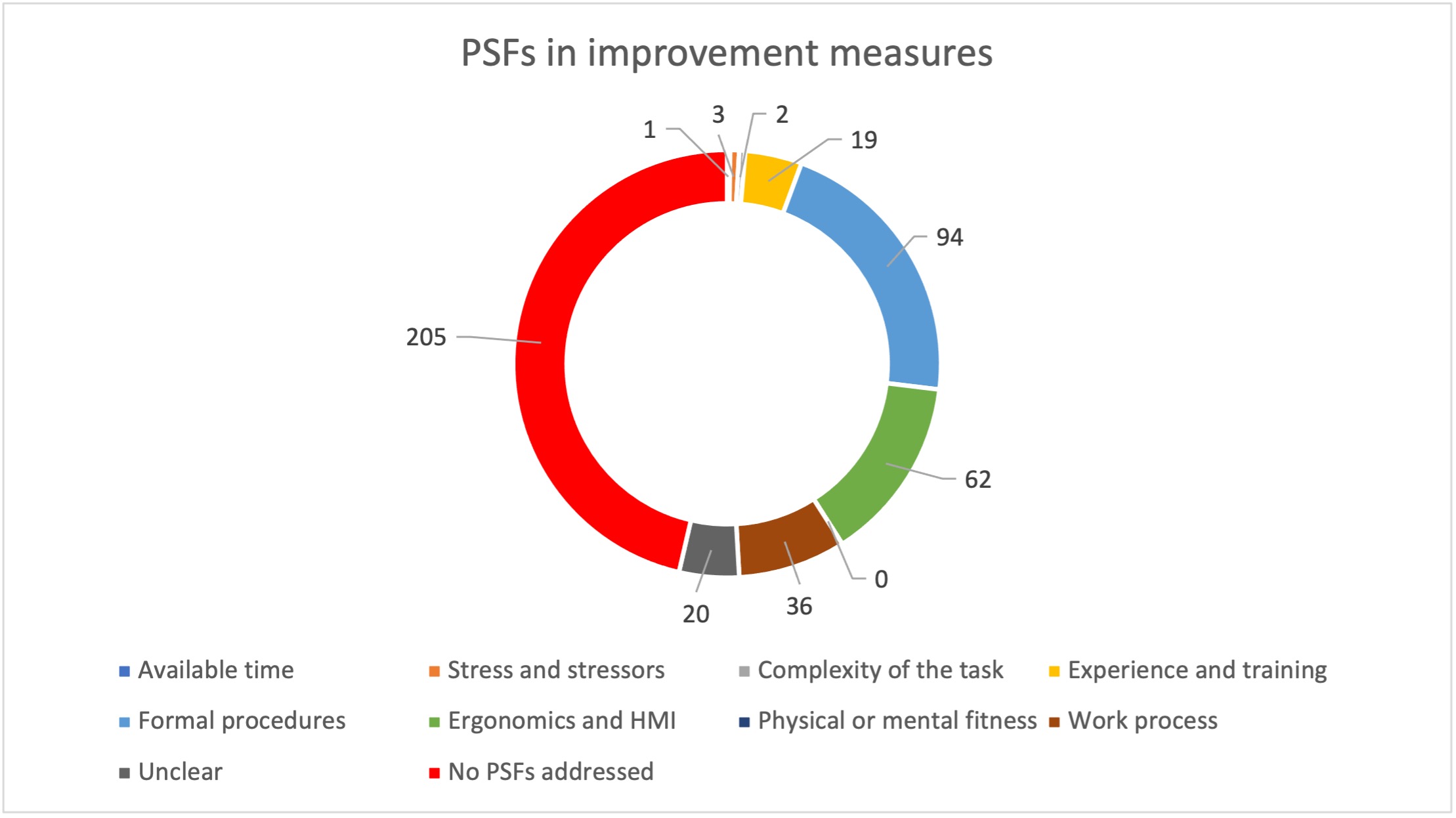

Supplement: znac067_Supplementary_Data [file znac067_supplementary_data.zip › Supplementary_Figure_1.jpg]
